# Visual measures of perceived roll tilt in pilots during coordinated flight and gondola centrifugation

**DOI:** 10.3233/VES-220016

**Published:** 2023-02-22

**Authors:** Arne Tribukait, Eddie Bergsten, Andreas Brink, Ola Eiken

**Affiliations:** a Department of Clinical Neuroscience, Section for Eye and Vision, Karolinska Institutet, Stockholm, Sweden; b Division of Environmental Physiology, Swedish Aerospace Physiology Center, Royal Institute of Technology, KTH, MTH, School of Chemistry Biotechnology and Health, Solna, Sweden

**Keywords:** Vestibular system, vestibular psychophysics, spatial disorientation, subjective horizontal, subjective vertical

## Abstract

**BACKGROUND:**

During a simulated coordinated turn in a gondola centrifuge, experienced pilots show a substantial inter-individual variability in visual measures of perceived roll tilt. Because of the centrifuge’s small radius, the pattern of stimuli to the semicircular canals during acceleration of the centrifuge differs in certain respects from that of an aircraft entering a turn.

**OBJECTIVE:**

To explore whether these differences may be of significance for the pilot’s roll- plane orientation and whether individual characteristics revealed in the centrifuge correspond to those during real flight.

**METHOD:**

8 fixed-wing air-force pilots were tested in a centrifuge and a high-performance aircraft. The centrifuge was accelerated to 2 G (gondola inclination 60°) within 10 s. The duration at 2 G was 6 minutes. Similar profiles were created in the aircraft. The subjective visual horizontal (SVH) was measured using an adjustable luminous line in darkness. Each pilot was tested on three occasions: centrifuge (2 runs), aircraft (2 turns), centrifuge (2 runs). For each 2-G exposure, initial and final SVH values were established via curve fitting.

**RESULT:**

Despite a large inter-individual variability (±SD), group means were similar in the aircraft (initial: 43.0±20.6°; final: 22.5±14.8°) and centrifuge (initial: 40.6±17.0°; final: 20.5±16.0°). Further, individual peculiarities in response patterns were similar in the two conditions. For both the initial and final SVH tilt there was a high correlation between centrifuge and aircraft.

**CONCLUSION:**

The correspondence between conditions suggests that the centrifuge is an adequate means for demonstrating the fundamental motion pattern of coordinated flight and also for establishing the individual pilot’s ability to perceive an aircraft’s roll attitude.

Findings are discussed in connection with vestibular learning and the possibility of underlying differences between pilots in the keenness for semicircular canal and somatosensory cues.

## 1 Introduction

One of the most basic flight manoeuvres is the co-ordinated turn, where the pilot adjusts the aircraft’s roll attitude so that the resultant gravitoinertial force vector remains parallel with the median plane of the body. Even if the entering of a turn is performed rapidly and results in a roll-plane stimulus to the semicircular canals [6, 31], a dominance of the graviceptive systems may lead to large under-estimations of the bank angle. Pioneering reflections on this vestibular dilemma had been published by the German ace Friedrich Noltenius, who identified the semicircular canals as a factor that may facilitate the maintenance of spatial orientation during curved flight [17]. However, few experiments have been performed to give a quantitative characterisation of this kind of spatial disorientation during real flight. Van Wulfften Palthe [30] asked blind-folded test subjects (who had flight experience) to give verbal estimates of the roll attitude during turns with bank angles of 45–75 degrees, entered within a few seconds. The responses were often largely erroneous. Tschermak and Schubert [28] used a visual indicator to obtain a quantitative measure of the perceived roll tilt during 2- G (60-degree) turns. The single test subject (who was also one of the authors) indicated a tilt of only 10 degrees. In spite of these early reports, few studies have been done with quantitative recording of the perceived horizontal plane during co-ordinated flight.

**Fig.1:**
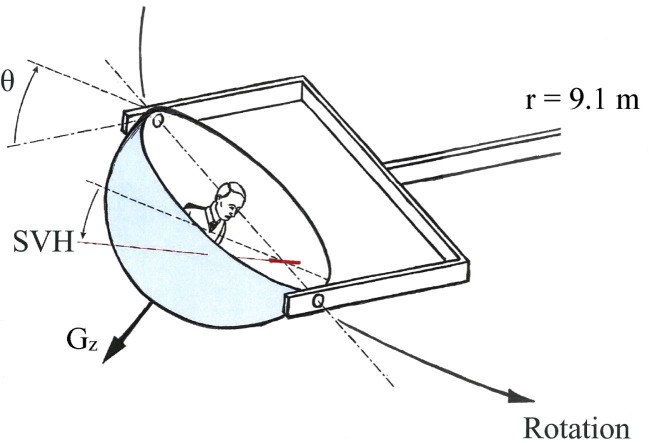
The gondola centrifuge. During acceleration, the cabin is rolled so that the resultant of the Earth gravity force and the centrifugal force remains aligned with the head-to-seat (z) axis of a subject sitting upright in the gondola. Thus, the graviceptive systems persistently signal that the head is upright in roll. Nevertheless, the change in roll position is an angular- *displacement* canal stimulus (which amounts to 60° as the resultant gravitoinertial force vector reaches the plateau value 2 G). In addition, because of the change in roll position, the angular- *velocity* stimulus, related to the rotation of the centrifuge about its main axle, gradually changes from yaw-left to near pitch backward. The yaw and pitch components are an order of magnitude greater in the centrifuge than during the corresponding G profile in an aircraft entering a co-ordinated turn.

It is generally assumed that the entering of a co-ordinated turn can be simulated in a large centrifuge with tangentially pivoted (swing-out) gondola [8, 12]. During acceleration of the centrifuge, a subject, seated-upright facing-forwards, experiences an increasing gravitoinertial force vector (vectorial sum of the Earth gravity force and the centrifugal force) that remains aligned with the subject’s head and body long (z) axis (Fig. 1). Therefore, the graviceptive systems persistently signal that the subject is upright in roll. However, the swing out of the gondola during acceleration is a roll-plane angular-displacement stimulus to the vertical semicircular canals [6, 8, 31]. In this respect, the stimulus situation in the centrifuge is similar to that encountered during a co-ordinated turn with an aircraft.

Nevertheless, there are certain notable differences between the stimulus pattern created in the centrifuge and that in an aircraft entering a turn. The centrifuge usually has a fixed radius and the increase in G force (and the gondola tilt) is attained via tangential acceleration. In an aircraft, maintaining constant speed, the G-level is determined by the radius of the trajectory. Further, the radius of a centrifuge is typically less than 10 metres, whereas the trajectory of an aircraft, making a 2-G turn at 500 km/h, will have a radius of 1137 metres. Consequently, the angular velocity (planetary rotation) will be approximately 10 times greater in the centrifuge than in the aircraft [27]. Thus, although the gondola centrifuge is regarded an important device for elucidating vestibular mechanisms of significance for the spatial disorientation problem in aviation, the above-mentioned differences motivate quantitative studies on how pilots perceive the roll tilt during centrifugation as well as in a corresponding turn with an aircraft.

In this connection, it might be worthwhile to summarize the sensory impressions that could, potentially, influence the pilot’s perception of roll position during a co-ordinated turn (or in the centrifuge). Firstly, the change in roll position is a stimulus to the vertical semicircular canals. In the central nervous system, the canal signal for angular velocity may be integrated over time, yielding a measure of roll angular *displacement* [14]. Secondly, the increasing roll tilt in combination with the planetary rotation (angular velocity of the aircraft with respect to the north-south direction, or of the centrifuge about its main axle) determines a *pattern* of angular velocity components in yaw and pitch, the yaw component dominating in the beginning whereas the pitch component will predominate when the roll tilt exceeds 45 degrees; therefore, the relative intensity of the yaw and pitch components could, in principle, be used as an indicator of the magnitude of roll tilt [25, 27]. Thirdly, although the otolith organs and somatosensory systems persistently signal that the pilot is upright in roll (thus apparently contradicting the canal message), during flight at constant altitude the magnitude of the G vector increases in a specific fashion with increasing roll tilt (i.e. the magnitude of the G force equals the inverse of the cosine for the roll angle; e.g. at 25 degrees the load is 1.1 G, at 60 degrees it is 2.0 G.). Therefore, as noted in earlier studies, it would be possible to estimate the bank angle via the sensation of increased weight [26]. However, this graviceptive clue does not tell the difference between a right-turn and a left-turn.

A measure of the perceived roll-tilt angle can be obtained by asking the subject to adjust a luminous line in darkness so that it appears to be horizontal (i.e. so that it corresponds with the subject’s spontaneous imagination of the horizon of the external world). This measure of spatial orientation is denoted the subjective visual horizontal (SVH). In the 1-g environment, healthy subjects, sitting upright, usually set the line with high precision close to the true gravitational horizontal [5]. Thanks to an otolith-dependent compensation process, the SVH remains approximately horizontal also during moderate static lateral head and body tilt [19].

An analogous measure, which has found extensive applications within the field of vestibular psychophysics, is the subjective visual vertical (SVV) [2, 15, 16]. Classical experiments, where the SVV was measured during different combinations of head tilt and G level, have elucidated the relationships between otolith stimulation and spatial orientation [18]. In more recent investigations a manual indicator was used to establish how subjective estimates of roll tilt are dependent on the G level, leading to insights into the central nervous processing of vestibular signals [4]. Both kinds of measurement indicate that the perceived roll tilt angle is greater in hypergravity than at 1 g. When it comes to the problem of spatial disorientation in aviation, however, the SVH would be a more adequate measure since the pilot’s external visual world is usually dominated by a horizon.

In the gondola centrifuge, acceleration from stationary to a pre-determined G level induces a sensation of being tilted towards the centre. This phenomenon can be studied via recording of the SVH. In non-pilots, the *initial* SVH tilt (i.e. the value in the beginning of the period with constant G elevation) is on average 30% of the gondola tilt, suggesting a considerable under-estimation of the bank angle. Further, during constant angular velocity of the centrifuge, the SVH tilt typically declines exponentially with a time constant of 1-2 minutes [21, 22]. Even though there are large differences between individuals, studies with repeated testing have confirmed that these differences are not due to random variation but reflect persistent individual characteristics in spatial orientation [20, 22]. Further, recordings with subjects seated in different directions in the gondola have revealed that the SVH tilt is not simply caused by the roll-plane angular *displacement* stimulus but that also the *pattern* of canal angular-*velocity* stimuli in yaw and pitch plays a significant role [21, 25, 26].

In pilots, the SVH response during centrifugation is typically greater and commonly does not decline with time, suggesting an effect of flight experience. Nevertheless, the inter-individual variability among pilots is considerable, but each individual shows little variation if tested at different occasions [20, 26]. The question arises whether the large inter-individual variability in the measure of perceived roll tilt during centrifugation corresponds to differences during real flight. Alternatively, the variability in the centrifuge could be a consequence of differences in stimulus pattern between aircraft and centrifuge. Namely, pilots might constitute a homogeneous group during real flight, but if some of them do not experience the stimulus situation in the centrifuge as flight-like, then the ability attained via flight experience would not be “transferred” to the centrifuge. This could, hypothetically, explain why SVH responses in the centrifuge represent a considerable degree of spatial disorientation as well as a large inter-individual variability. This issue can only be resolved by testing a heterogeneous group of subjects during real flight-turns as well as during centrifugation.

In a recent study with non-pilots, the SVH was recorded during gondola centrifugation (G level 1.56, gondola inclination 50°) and during co-ordinated turns with a propeller aircraft (G level 1.4, roll tilt approximately 44°). The SVH responses, corrected for minor differences in the roll tilt stimulus, were very similar in the two conditions. There were substantial inter- individual differences, but for each individual, data obtained in the aircraft corresponded to those obtained during centrifugation [23]. However, the quantitative and qualitative differences between non-pilots and pilots, revealed in several earlier centrifuge studies, warrants further investigations of this kind, with pilots as test subjects.

The primary aim of this study was to investigate whether the SVH during gondola centrifugation can be regarded a truthful measure of the pilots’ ability to perceive the roll position also during real co-ordinated flight. This aim resolves itself into two parts. On the one hand, there is the question whether a simulated co-ordinated turn in a centrifuge is experienced by pilots as flight-like. On the other hand, it is desirable to clarify whether testing in the centrifuge can reveal individual characteristics in spatial orientation that are significant also during real flight.

## 2 Methods

### 2.1 Subjects

Eight healthy males (denoted A-H), aged 24–27 years, who had recently finished the tactical flying training for fighter pilots in the Swedish Air Force, were recruited for the study. All subjects had 270 flight hours in a twin-engine jet trainer (Saab Sk60), including advanced aircraft training, air combat manoeuvres, formation flying and tactical low-level navigation.

Three of them (E, F and H) had earlier completed high-school education for commercial pilot license and had, respectively, 140, 500 and 120 flight hours in small propeller aircraft.

Further, all pilots had undergone G-training in the centrifuge and were familiar with this device; they were aware of its dimensions and direction of rotation and knew that the gondola is tilted during centrifugation so that the subject always remains upright with respect to the resultant gravitoinertial force vector. They had also been informed that in the present study the aircraft experiment would be analogous to the experiment in the centrifuge, comprising left-turns with similar G load. However, the precise G load was not revealed to the subjects; they were informed that it would be between 1.5 and 3 G.

### 2.2 Study design

Each subject participated in three experimental sessions, performed in the following sequence: (i) centrifuge session 1, (ii) in-flight experiment, (iii) centrifuge session 2. Each centrifuge session comprised two 2-G runs with durations of 6 min; the in-flight experiment comprised 2 left-turns with the same G load and duration as the centrifuge runs. As a rule, the interval between centrifuge occasion 1 and the in-flight experiment was 2 weeks; exceptions were subject B and G (interval 12 weeks) and subject C (interval 1 day). Occasion 2 in the centrifuge took place a few hours after the in-flight experiment.

### 2.3 Equipment and general procedures

#### 2.3.1 Centrifuge

The centrifuge experiments were performed in the dynamic flight simulator (Wyle Laboratories Inc., El Segundo, CA) at the Aviation Physiology Laboratories in Linköping. This centrifuge has a radius of 9.1 m. Its rotation is anticlockwise (as seen from above).

Facing forwards in the gondola, the subject was fixed in a cockpit seat by means of safety belts. The head was not restrained (as this would not be allowable in the aircraft) but the subject was instructed to avoid head movements by keeping the back of the head against a head rest. The rest had a u-shaped vertical groove for the back of the head and was padded with 1 cm of foam rubber. It was adjustable in the vertical and antero-posterior directions, making it natural and comfortable for each subject to keep the head stationary in the upright position during the entire recording periods.

The roll position of the gondola was computer-controlled so that the resultant gravitoinertial force vector was always parallel with the subject’s median plane. In addition, during acceleration and deceleration of the centrifuge the pitch position of the gondola was adjusted with respect to the tangentially acting inertial component; thus, also in pitch, the orientation of the subject did not change with respect to the resultant gravitoinertial force vector. Planetary acceleration and deceleration was 7.8°/s^2^. From stationary the 2-G level was attained in 10 s. During the experiments the gondola was completely darkened but the subject was observed in infrared light by means of a video camera and could always communicate with the experimenter via a two-way intercom system.

#### 2.3.2 Aircraft

The in-flight experiments were carried out in a high-performance jet trainer (Saab Sk60). The two standard catapult chairs had been replaced by simple seats, giving place for two extra seats behind. Pilot flying (an experienced fighter pilot) was sitting in the left front seat with the experimenter to the right. The test subject was seated in the left rear seat. To the right of him was a technician managing data collection. All wore headsets and could communicate freely with each other. The subject stabilised his head against a head rest, similar to that used in the centrifuge. In addition, securing the head to the face-mask device (see below) ensured that the sagittal plane of the head was persistently aligned with the long axis if the aircraft and that rotational movements of the head were minimized.

The air speed was 290–310 kn. The pilot aimed at entering, in a coordinated manner, left turns with a bank angle of 60° (2 G) within 10 sec and to maintain each turn for 6 min. During the turns, the pilot’s manoeuvring was based primarily on the attitude indicator and instruments presenting the G level and any deviation between the G vector and the aircraft’s vertical plane. By slowly increasing the altitude, we avoided the vibrations which can occur when passing through the vortex of the aircraft’s own trajectory. Exiting the turns was also performed in 10 sec and with the resultant G vector persistently in the head-to-seat direction. The resulting trajectory would ideally be circular with respect to the air, not as projected on the ground. As a rule, each turn was preceded and followed by at least 2 min of straight-ahead level flight.

Complete darkness was created in the following way. The test subject wore a modified diver’s mask, the glass of which had been removed. The mask was connected to the device with the luminous line via a light-proof flexible tube (diameter 20 cm), consisting of an external layer of reflecting plastic and aluminium foil and an internal layer of black velvet. Light-proof ventilation channels permitted breathing through the nose. The subject could put on and remove the mask and the head set without assistance.

Resultant Gz level and roll attitude were recorded with a frequency of 5 Hz by means of a 3DM-GX3-45 Miniature GPS-Aided Inertial Navigation System (LORD Microstrain®, Williston, VT). This device also provided a recording of the aircraft’s trajectory relative to the surface of the Earth. In addition, the instruments were continually observed by the experimenter who also made notes on relevant indications.

#### 2.3.3 Recording of the SVH

In front of the subject (at eye level and straight ahead, 50 cm from the subject’s eyes) there was a line of red light-emitting diodes, 75 mm long and 1.7 mm wide, mounted on the axle of a digital servo (DSR 1015, Thunder Tiger Corp, Taichung City, Taiwan). The axis of rotation coincided with the subject’s naso-occipital (visual) axis. The servo was controlled by a microprocessor (Arduino, UNO with a program in C). Every time the line was switched on, the subject adjusted it, using two push-buttons on a remote control, so that he perceived it as horizontal (i.e. so that it corresponded with his spontaneous imagination of the horizon of the external world). If the subject kept one of these buttons pressed, the rotation of the line was 11°/s; by briefly tapping the buttons the subject could adjust the orientation of the line in steps of 0.2°. When pleased with a setting the subject pressed a third button, which extinguished the line. With an accuracy of 0.1° the deviation of the line from the gravitoinertial horizontal (i.e. the transversal plane of the gondola or aircraft) was automatically recorded. It was then instantaneously offset 8–26° (randomly), alternately clockwise and counter-clockwise with respect to the subject’s latest setting, and switched on again after a latency of 1 s.

Programming for the recording of data from the microprocessor was performed in LabView (National Instruments Corporation, Austin, TX) on a HP ProBook 6570 (Intel Core i5, 2.60 GHz) connected to the microprocessor via a network cable.

As rule, recording of the SVH commenced at least 1 min before acceleration of the centrifuge or the entering of a turn with the aircraft. After deceleration of the centrifuge, and after the exiting of a turn with the aircraft, recording proceeded for at least 2 min at 1 G.

### 2.4 Definitions and treatment of data

Tilts of the SVH to the right (clockwise from the subject’s point of view) are denoted positive; tilts to the left are denoted negative. Thus, a true response to the roll tilt during a left-turn would have a positive sign. For each centrifuge run and aircraft turn, the 1-g value was calculated as the mean of the settings obtained during the 1-min period preceding acceleration (centrifuge) or the beginning of a turn (aircraft). As regards the 2-G plateau, time zero is defined as the point in time where the 2-G plateau (and constant angular velocity) was attained. For the flight experiments, the point in time when the entering of a curve commenced, as well as when the entering was complete (corresponding to t = 0 in the centrifuge experiments) was established by scrutiny of recordings of Gz and the aircraft’s roll position.

In contrast to non-pilots, at the 2-G plateau pilots rarely show a time course for the SVH that can be characterised by simple exponential decay function. Therefore, in accordance with earlier studies on pilots [20, 27] the initial SVH tilt at 2 G was obtained by means of linear curve-fitting to the data points from the 1^st^ min at 2 G and extrapolation to t = 0. As regards the final deviation, it can be assumed that the SVH has stabilized after three min. Thus, the final deviation was calculated as the mean of the data points obtained during the last 2 min at the 6-min plateau. In the aircraft, the duration of the 2-G plateau often deviated from the stipulated 6 min; the pilot had to exit the turn at a point permitting a 2-min straight-ahead level flight without transgressing the borders given by the control tower. Therefore, to make the data from the flight experiments match those obtained in the centrifuge, the following principles were applied: If the duration of the 2-G plateau happened to be shorter than 6 min, the interval for calculating the final deviation was reduced by the same amount. If it were longer, settings made during the excessive time span were not included in the calculation of the final deviation.

To compare the two conditions (centrifuge vs. aircraft) with respect to initial and final SVH tilt, data were treated as follows: Initial and final SVH tilts were obtained for each centrifuge run. For each centrifuge occasion the individual means for run 1 and run 2 were calculated. From these we calculated the overall means for occasion 1 and 2. Using paired *t*-tests, the overall means for the centrifuge were compared with the means for turn 1 and 2 in the aircraft. For evaluating the correspondence between results obtained in the two conditions, linear regressions analyses were done for initial and final deviations.

As regards individual characteristics, in addition to the initial and final SVH tilts per se, the decline with time at the 2-G plateau (defined as the initial tilt minus the final) was analysed, as well as the subjects’ precision (or variability) in the settings with the line (defined as 1 SD for the 1-g value, the RMS error for the initial deviation and 1 SD for the final deviation).

## 3 Results

### 3.1 SVH at 1g

In the centrifuge, subjects made on average 14.3 settings (SD = 3.6) per min with the line. The number of settings was similar in the aircraft (13.5±3.7). When the subjects were sitting upright at 1 g, the SVH did typically not deviate by more than 3 degrees from the true gravitational horizontal. Thus, in the centrifuge, the overall group mean (based on individual means for occasion 1 and 2) was –1.41±1.25°. In the aircraft it was –0.38±2.44°. In the centrifuge, most subjects had a variability in the settings (expressed as 1 SD) that was less than 1.0°; the overall group mean was 0.91±0.42°. It was slightly larger in the aircraft: 1.40±0.67°.

### 3.2 SVH at 2 G, qualitatively

As a rule, acceleration of the centrifuge to 2 G induced a tilt of the SVH to the right (clockwise as seen by the subject), corresponding to a sensation of head and body tilt to the left.

**Fig.2a-h:**
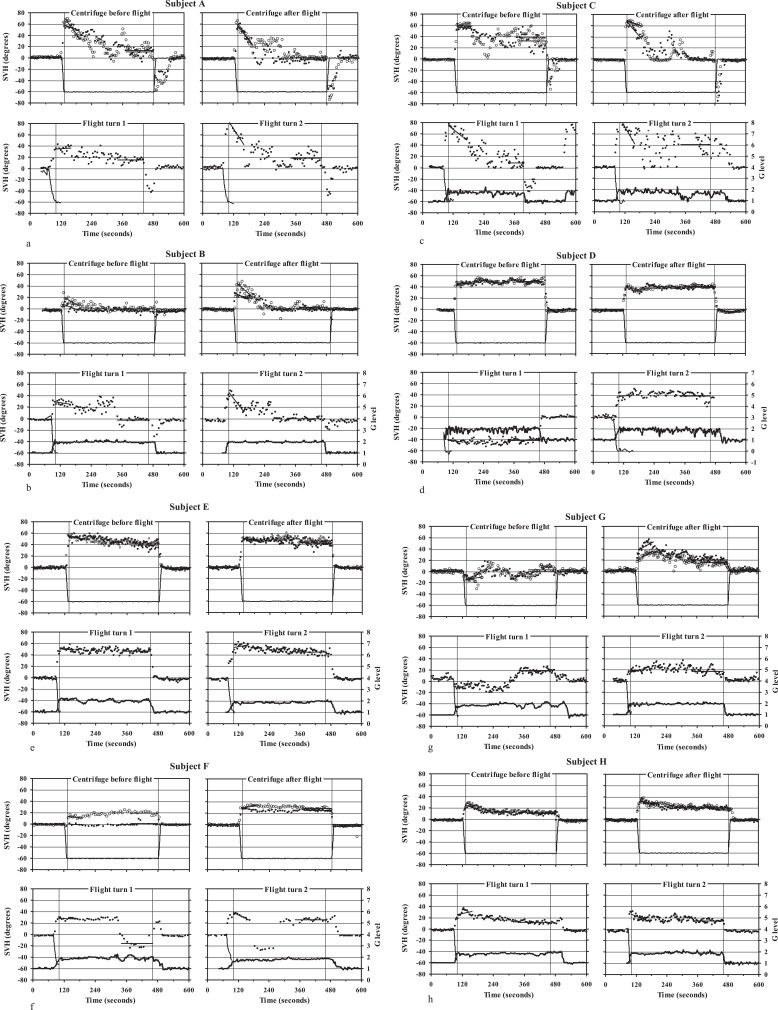
Individual data for the SVH obtained in the gondola centrifuge and aircraft. Each data point represents one setting with the luminous line. At each centrifuge session the subject underwent two runs (run 1: black symbols; run 2: open symbols). Initial and final SVH values (indicated by lines) were obtained via linear curve fitting to data points from the first minute at 2 G and as the mean for the last two minutes. Thin curves represent recorded roll position of the centrifuge gondola or aircraft. In the aircraft experiments, a roll signal could only be obtained for the beginning of each turn. Bold curves represent the resultant G level recorded in the aircraft (secondary y axis). In a few cases, one of these signals was lost because of technical malfunction. Subject A, B and C displayed a pattern characterised by a decline in the SVH tilt during the 2-G exposure and an after-effect in conjunction with centrifuge deceleration and when the aircraft reassumed straight-ahead flight. In Subject D, E and F the SVH tilt tended to remain at a similar value during the 2-G exposure. In these cases there was no after-effect. However, it occurred that these subjects made errors in the direction of roll tilt (Subject D) or showed sudden reversals in the direction of indicated roll tilt during the 2-G exposure (Subject F). Subject G and H showed less pronounced response patterns. In spite of individual peculiarities, there was a conspicuous correspondence between data from the centrifuge and aircraft.

Differences between subjects were considerable. However, as can be seen in Fig. 2a-h, at 2 G each subject tended to display a similar pattern in the aircraft as in the centrifuge. For the sake of clarity, findings will first be described qualitatively, with subjects tentatively categorised into three groups according to general response characteristics.

One rather obvious pattern, displayed by group 1 (subjects A, B and C), consisted in a tilt of the SVH that was of great magnitude in the beginning of the 2-G plateau but declined substantially with time. In these cases, deceleration of the centrifuge, or exiting turns with the aircraft, typically induced an after-effect with SVH tilts in the opposite direction (Fig. 2a-c). The other noticeable pattern (group 2) was a tilt of the SVH that remained approximately constant during the entire 2-G plateau. In these subjects (D, E and F) no after-effect was seen in conjunction with deceleration of the centrifuge or exiting of the aircraft turns (Fig. 2d-f).

The two subjects of group 3 (G and H) showed less pronounced or intermediary patterns (Fig. 2g,h).

In group 2 and 3 there were instances where the subject indicated a pronounced roll tilt but misperceived the *direction* of the turn, hence adjusting the line as if he was in a right-turn (although the direction of centrifuge rotation was counter clockwise and the aircraft always made left-turns). For instance, subject G (Fig. 2 g) reported that he had experienced the centrifuge runs at occasion 1, as well as the first minutes of turn 1 in the aircraft, as right- turns. In contrast, during the second turn with the aircraft, and in the centrifuge runs at occasion 2, he always felt as if being in a left-turn. Subject D (Fig. 2d) was very consistent in the *magnitude* of indicated tilt, both in the centrifuge and during flight. The SVH tilt was substantial and did not decline with time, but during turn 1 in the aircraft he persistently adjusted the line as if experiencing a right-turn. During turns with the aircraft, subject F (Fig. 2f) showed a few abrupt transitions in the direction of the SVH tilt, as if the persistent G load told him that he was in a coordinated turn but that this clue was ambiguous when it comes to the *direction* of the turn.

### 3.3 SVH at 2 G, quantitatively

Individual values for the initial and final deviation are shown in Table 1 and 2. Cases where the subject has adjusted the line as if experiencing a right-turn have not been included in group statistics. In addition, during the first centrifuge run at occasion 1, subject F (Fig. 2f), due to a misunderstanding, performed the *egocentric* task of adjusting the line so that it was parallel with the transversal plane of his own body (yielding SVH values close to zero), although he verbally confirmed a sensation of being tilted to the left with respect to the outer world. In the pause, it was clarified that the task was to indicate the perceived horizon of the external world, not the perceived horizontal of the gondola or the own body. Data from the first centrifuge run of subject F are therefore not included in statistics at group level.

**Table 1:** Initial SVH tilts at the 2-G plateau in the centrifuge and during left-turns with the aircraft. Values (in degrees) were obtained by means of linear curve fitting to data points from the first minute at the 2-G exposure. R1, run 1; R2, run 2; T1, turn 1; T2, turn 2; M, mean for R1 and R2 (or T1 and T2); OM, overall mean in the centrifuge. Values within parenthesis are not included in group statistics (see text)

Subject No.	Initial deviation of the SVH at 2 G - Centrifuge							Initial deviation of the SVH at 2 G - Aircraft		
	Occasion 1			Occasion 2			OM
	R1	R2	M	R1	R2	M		T1	T2	M
A	57.9	70.8	64.4	60.9	60.1	60.5	62.4	34.4	84.0	59.2
B	7.2	20.1	13.6	23.6	44.7	34.1	23.9	28.8	46.7	37.7
C	57.5	60.0	58.8	67.2	68.3	67.8	63.3	76.9	88.6	82.8
D	42.3	46.7	44.5	39.3	40.6	40.0	42.2	(–41)	36.7	36.7
E	52.3	56.3	54.3	49.6	50.3	49.9	52.1	48.6	52.9	50.8
F	(0.3)	13.9	13.9	28.2	28.9	28.5	21.2	27.7	38.5	33.1
G	(–13.0)	(–14.1)	(–13.6)	21.3	37.7	29.5	29.5	(–12.6)	18.4	18.4
H	25.0	28.7	26.8	34.2	32.2	33.2	30.0	25.5	26.0	25.7
**Mean**	**40.4**	**42.4**	**39.5**	**40.5**	**45.3**	**42.9**	**40.6**	**40.3**	**49.0**	**43.0**
**1 SD**	**20.4**	**21.7**	**21.3**	**17.1**	**13.6**	**14.8**	**17.0**	**19.8**	**25.5**	**20.6**

**Table 2:** Final SVH tilts at the 2-G plateau during centrifugation and left-turns with the aircraft. Values (in degrees) are means of data points obtained during the last 2 minutes of the 2-G exposure. R1, run 1; R2, run 2; T1, turn 1; T2, turn 2; M, mean for R1 and R2 (or T1 and T2); OM, overall mean in the centrifuge. Values within parenthesis are not included in group statistics (see text)

Subject No.	Final deviation of the SVH at 2 G - Centrifuge							Final deviation of the SVH at 2 G - Aircraft		
	Occasion 1			Occasion 2			OM
	R1	R2	M	R1	R2	M		T1	T2	M
A	10.1	13.1	11.6	–3.1	0.2	–1.4	5.1	15.2	18.5	16.8
B	–4.1	0.2	–2.0	–0.9	1.7	0.4	–0.8	–2.8	–0.4	–1.6
C	31.8	37.4	34.6	–0.8	0.3	–0.3	17.2	8.0	40.4	24.2
D	48.2	49.3	48.8	38.7	40.8	39.7	44.2	(–41.8)	38.6	38.6
E	39.4	42.7	41.0	42.2	46.1	44.1	42.6	46.2	45.0	45.6
F	(0.6)	19.2	19.2	24.1	27.5	25.8	22.5	(–15.6)	25.6	25.6
G	(–0.2)	(2.6)	(1.2)	15.1	20.3	17.7	17.7	18.2	17.0	17.6
H	10.8	11.5	11.2	19.6	20.0	19.8	15.5	11.7	14.6	13.2
**Mean**	**22.7**	**24.8**	**23.5**	**16.9**	**19.6**	**18.2**	**20.5**	**16.1**	**24.9**	**22.5**
**1 SD**	**20.2**	**18.4**	**18.4**	**17.8**	**18.0**	**17.9**	**16.0**	**16.4**	**15.5**	**14.8**

**Fig.3:**
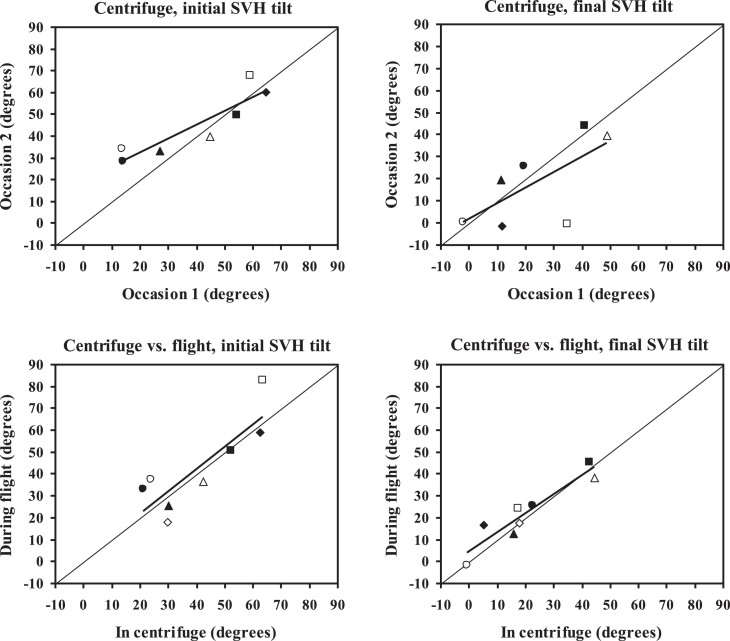
Correspondence between SVH values obtained at the 1^st^ and 2^nd^ test occasions in the centrifuge (upper diagrams) and between values obtained in the centrifuge and aircraft (lower diagrams). Each data point represents one individual. Each individual is represented by the same symbol in all diagrams. Diagonal lines represent ideal correspondence, bold lines the outcomes of linear regression analysis.

In the centrifuge, the initial deviation of the SVH was 39.5±21.3° (*n* = 7, mean for run 1 and 2 at occasion 1) and 42.9±14.8° (*n* = 8, mean for run 1 and 2 at occasion 2). The overall mean was 40.6±17.0° (*n* = 8). There was a high correlation between data from occasion 1 and 2: (linear regression) r = 0.90, *p* = 0.005, *n* = 7 (Fig. 3, upper left diagram). In the aircraft, the initial deviation was 43.0±20.6° (*n* = 8, mean for turn 1 and 2). There was a high correlation between data obtained in the centrifuge and those from the aircraft experiments: r = 0.85, *p* = 0.008, *n* = 8 (Fig. 3, lower left diagram).

In the centrifuge, the final deviation was 23.5±18.4° (*n* = 7, mean for run 1 and 2 at occasion 1) and 18.2±17.9° (*n* = 8, mean for run 1 and 2 at occasion 2). The overall mean was 20.5±16.0° (*n* = 8). There was a tendency to correlation between data from the two occasions: r = 0.67, *p* = 0.10, *n* = 7 (Fig. 3, upper right diagram). In the aircraft, the final deviation was 22.5±14.8° (*n* = 8, mean for turn 1 and 2). There was a high correlation between data from the centrifuge and those obtained in the aircraft: r = 0.94, *p* = 0.0005, *n* = 8 (Fig. 3, lower right diagram).

Table 3 shows the after-effect as related to the decline of the SVH with time at 2 G. For each centrifuge run and aircraft turn a measure of the after-effect, which as a rule was short-lasting, was defined as the greatest average deviation of three consecutive data points (running average) obtained during or immediately following deceleration (or exit of turn).

**Table 3:** Subjects who displayed a pronounced decline in the SVH tilt during the 2-G exposure often responded with a large but short-lasting after-effect upon deceleration of the centrifuge and when the aircraft reassumed straight ahead flight after being in a left-turn. The luminous line was thus set as if the subjects experienced a rightward banking (negative values)

Subject No.	SVH - decline with time at 2G						SVH - after-effect at 1g					
	In centrifuge				In aircraft		In centrifuge				In aircraft	
	Occasion 1		Occasion 2				Occasion 1		Occasion 2	
	R1	R2	R1	R2	T1	T2	R1	R2	R1	R2	T1	T2
A	47.8	57.7	63.9	59.9	19.2	65.5	–35.7	–55.0	–58.0	–70.3	–39.6	–45.9
B	11.3	19.9	24.5	43.0	31.6	47.0	–11.3	0	–15.5	0	–25.6	–15.2
C	25.7	22.7	68.0	68.0	68.9	48.3	–38.4	–45.3	–65.4	–54.5	–39.4	0
D	–5.9	–2.5	0.6	–0.1	–	–1.9	0	0	0	0	–	0
E	12.9	13.6	7.4	4.2	2.5	7.9	0	0	0	0	0	0
F	–	–5.3	4.1	1.4	–	12.9	–	0	0	0	–	0
G	–	–	6.2	17.4	–	1.4	–	–	0	0	–	0
H	14.2	17.1	14.6	12.2	13.8	11.4	0	0	0	0	0	0

Table 4 shows the variability in the settings with the line for each of the main variables in the centrifuge and aircraft. During ideal (baseline) conditions, i.e. at 1 g in the centrifuge, differences between individuals may to some extent be explained by the fact that each subject performed the task at his own pace, ranging between 8.5 and 20 settings per minute. Thus, at 1 g in the centrifuge there was a correlation between the variability and the number of settings per minute: r = 0.87, 0.005 (linear regression, *n* = 8). Except for this basic observation, it can be noted that the group means for the variability was slightly higher in the aircraft than in the centrifuge. Further, it was higher at 2 G than at 1 g and higher for the final deviation than for the initial deviation. The highest values for the variability were displayed by subjects in whom the initial deviation was large in comparison with the final deviation. Nevertheless, for any of the main variables (SVH at 1 g as well as the initial and final deviation at 2 G) the variability did not correlate significantly with the magnitude of tilt, i.e. there was no general tendency of the variance to increase with increasing SVH deviation. In addition, although the initial SVH deviation was twice as large as the final deviation, it was not associated with a higher variability.

**Table 4:** Intra-individual variability in the settings of the luminous line in relation to SVH values obtained at 1 g as well as during 2-G exposures in the centrifuge and aircraft. It can be noted that the variability was slightly larger in the aircraft than in the centrifuge. In both conditions the variability was greater for the final deviation than for the initial deviation. The variability did not correlate with the magnitude of the SVH tilt. The largest values of the variability were found among the subjects who had a large initial deviation but a substantial decline during the 2-G period (subject A, B and C). Conversely, in subjects who maintained a stable SVH tilt during the 2-G exposure (D, E and F), the variability tended to be smaller

Subject No.	Values obtained at 1g				Initial deviation at 2G				Final deviation at 2G			
	Centrifuge		Aircraft		Centrifuge		Aircraft		Centrifuge		Aircraft	
	SVH	SD	SVH	SD	SVH	RMS	SVH	RMS	SVH	SD	SVH	SD
A	–0.7	0.8	1.4	1.6	62.4	4.9	59.2	6.0	5.1	5.8	16.8	7.2
B	–2.9	0.9	–3.0	1.0	23.9	4.8	37.7	5.0	–0.8	2.9	–1.6	3.9
C	–1.9	0.5	0.3	1.0	63.3	3.8	82.8	6.5	17.2	7.7	24.2	11.2
D	–2.5	0.6	2.0	1.9	42.2	2.2	36.7	4.8	44.2	2.7	38.6	6.8
E	–0.8	1.3	–1.8	1.3	52.1	2.8	50.8	4.0	42.6	4.4	45.6	3.2
F	–1.5	0.5	–2.2	0.6	21.2	1.7	33.1	1.6	22.5	1.9	25.6	2.5
G	1.0	1.7	3.3	2.7	29.5	4.1	18.4	3.3	17.7	4.9	17.6	4.8
H	–2.1	1.0	–2.9	1.1	30.0	2.5	25.7	4.5	15.5	2.3	13.1	2.5
**Mean**	**–1.4**	**0.9**	**–0.4**	**1.4**	**40.6**	**3.4**	**43.0**	**4.5**	**20.5**	**4.1**	**22.5**	**5.3**
**SD**	**1.3**	**0.4**	**2.4**	**0.7**	**17.0**	**1.2**	**20.6**	**1.5**	**16.0**	**2.0**	**14.8**	**3.0**

## 4 Discussion

### 4.1 Essentials of the findings

#### 4.1.1 The magnitude of indicated roll tilt

During a co-ordinated 2-G turn (bank angle 60 degrees), pilots with similar education and flight experience showed a substantial inter-individual variability in the visual measure of perceived roll tilt; the range was approximately 40 degrees. In addition, there was a striking correspondence between data obtained in the gondola centrifuge and during co-ordinated flight. For both the initial and final SVH tilt there was a high and significant correlation between data from the centrifuge and aircraft, i.e. each individual tended to show similar values in the two stimulus situations. Considering group means and inter-individual variability the present values are compatible with those of earlier studies on experienced fighter pilots and military helicopter pilots [26]. This suggests that the basic flight education might have a relatively great impact on learning processes associated with curved flight.

#### 4.1.2 Response patterns

An additional observation was that the pilots did not simply differ with respect to the *magnitude* of indicated tilt. In spite of the small number of subjects, the study design, with repeated 2-G exposures and a great number of data points, made it possible to discern two principal response patterns. One of these is characterised by a tilt of the SVH which is large in the beginning of the 2-G exposure but then declines, approaching zero by the end of the 2-G exposure. In these cases there was, as a rule, an after-effect, with negative values in conjunction with deceleration of the centrifuge or exiting the turn with the aircraft. Presumably, these subjects responded to the canal stimuli associated with acceleration and deceleration of the centrifuge as well as with the entering and exiting of turns with the aircraft. The decline during the 2-G plateau would reflect the fading memory trace of the initial canal stimuli. The after-effect seems to be a counterpart to the so-called leans, i.e. an illusion of banking that can occur during straight-ahead and level flight after a rapid correction for a turn, entered involuntarily and slowly [3]. In contrast to common characterisations of the leans, the present after-effects were short-lasting, although the magnitude could be as large as the SVH tilt in the beginning of the preceding 2-G period.

The second major pattern consisted in a tilt of the SVH that remained at a similar magnitude during the entire 2-G exposure. No after-effects were seen in these cases, but there was another conspicuous phenomenon, namely errors - and even sudden reversals - in the direction of the SVH tilt. As discussed below, although pilots may associate the sensation of increased G load with being in a coordinated turn, the somatosensory input does not reveal the direction of the turn. Therefore, pilots who tend to rely on this information may experience an ambiguity in the perceived *direction* of banking.

### 4.2 Inter-individual variability and differences between pilots and non-pilots

#### 4.2.1 Characteristics of non-pilots

In non-pilots, acceleration of the gondola centrifuge typically induces a tilt of the SVH that initially approximates 30–40 per cent of the real gondola inclination and then declines exponentially with a time constant of 1-2 minutes, approaching an asymptote that rarely deviates more than 5 degrees from zero. Further, there is a considerable inter-individual variability; at 2 G the initial SVH tilt may range from near-zero to more than 40 degrees [22, 23]. A suggested explanation for the general underestimation of the roll tilt angle is that the graviceptive systems, which during gondola centrifugation persistently signal that the subject is upright in roll, contradict the semicircular canal response to the swing out of the gondola [7, 22, 29]. Accordingly, one factor behind the inter-individual variability could be that subjects differ with respect to the relative dependence on input from the semicircular canals and otolith organs.

#### 4.2.2 Characteristics of pilots

At group level, experienced pilots differ from non-pilots in three major ways [26]. Firstly, a considerable fraction of pilots show responses that are greater than in the most extreme non-pilots; SVH tilts approximating the real tilt of the gondola are frequent among pilots. Secondly, it is common that the SVH tilt does not decline with time. Thirdly, also among experienced pilots there is a great inter-individual variability; the final SVH tilt may range from near-zero to values approximating the gondola inclination.

#### 4.2.3 Reproducibility.

Studies with repeated testing have shown that differences between subjects are not due to uncertainties in the measurement procedure. In non-pilots, tested with an interval of 1-2 weeks, the reliability coefficient for the initial and final deviation was, respectively 0.86 and 0.87; the corresponding values for student pilots were 0.97 and 0.98 [20]. Also in the present material, there was a high correspondence between data from the two centrifuge occasions, in most cases separated by several weeks. Thus, it must be assumed that the inter-individual variability in the visual measure of perceived roll tilt represents true and comparatively stable individual characteristics.

### 4.3 The potential role of vestibular learning in pilots

The sense of balance is often regarded as a “silent companion”; spatial orientation and postural control are normally maintained via neural processing at a level not accessible to conscious scrutiny. This restricts the possibilities to gain insight into vestibular mechanisms via verbal reports by test subjects. As regards the inter-individual variability among non-pilots we have not received any verbal accounts from our test subjects that could serve as guidance in the search for an explanation. Thus, the above-mentioned hypothesis that some subjects are prone to utilise canal input whereas others rely mainly on the otoliths appears difficult to evaluate via introspection and verbalisation.

Things may be different when it comes to persons who have spent much time and attention on activities where the requirements on the vestibular system deviate from those of ordinary life. Learning complex motor skills may alert a person to cues or sensory qualities that do not usually reach conscious level. It is tempting to interpret the differences between pilots and non-pilots as an effect of flight experience. Entering a co-ordinated turn is one of the most basic and common flight manoeuvres. In due course, the visual impression of an external horizon would lead to an improved ability to interpret the complex vestibular input and the somatosensory impression of increased G load.

#### 4.3.1 The bodily sensation of increased weight

In earlier studies, several pilots have described that they associate the sensation of increased bodily weight and strain in the head-to-seat direction with being in a co-ordinated turn - and that an increase in G level is often accompanied by the visual impression of an external horizon, tilted with respect to the own transversal plane [20, 26]. Similar statements - and “pilot-like” SVH responses - have been made also by non-pilots with proficiency in motorcycle riding or certain sports activities [27].

Given the fundamental role of graviception, it might seem paradoxical that an increase in the magnitude of the G vector, acting in the head-to-seat direction, would lead to a sensation that this G vector is not pointing “downwards”. Nevertheless, analogues of co-ordinated flight are common in animal life. Running along a curved path would not be compatible with postural control if the perceived direction of the resultant G vector were taken as a representation of the Earth gravity field. Rather, the constancy of the Earth gravity field combined with the equivalence between gravity and inertia entails that a neural representation of the magnitude of the G vector may facilitate the task of attaining an adequate combination of speed, trajectory and lateral body tilt.

Further, during gondola centrifugation the decline in the SVH tilt among non-pilots occurs with a greater time constant, i.e. more slowly, at higher G levels [22]. Thus, although the gravitoinertial force vector, acting in the head-to-seat direction, is in conflict with the initial canal message (which induces the sensation of roll tilt) the increase in the G vector seems to promote the *memory* of the canal message. Also this observation suggests that the tendency in pilots to associate an increased G load with a bank angle has a natural basis and would not require any radical or “unnatural” re-interpretation of vestibular and somatosensory input.

#### 4.3.2 Spatial orientation learning during flight

Since the persistence of the SVH tilt is common in pilots, it must be assumed that the development of this phenomenon is driven by consequences of practical significance (positive feedback); an improvement in spatial orientation would reduce the cognitive workload of flying. But how could this be reconciled with the large inter-individual variability in the magnitude of the SVH tilt? One possibility is that pilots simply differ in constitutional properties underlying the development of a sense for the relationship between G load and bank angle. Alternatively, under ordinary flight conditions the value of this sense would not depend so much in *quantitative* accuracy. Even if not providing an approximate measure of the bank angle, it might support situational awareness in a *qualitative* way by maintaining the experience of being in a turn.

However, since the increase in G level *per se* cannot serve as a clue regarding the *direction* of banking, the value of an ability to translate G level into bank angle would be dependent on other sources of information, e.g. the semicircular canals. Thus, if the entering of a turn occurs slowly, or in case vibrations interfere with the pilot’s ability to detect the canal stimuli, then the G-dependent mechanism for roll-tilt perception may yield a direction error.

The occurrence of two distinct response patterns suggests that inter-individual variability is not merely a matter of quantity but that individuals may differ also with respect to the sensory modality underlying perceived roll tilt during co-ordinated flight. Pilots in whom the SVH tilt is large in the beginning of the 2-G exposure but then declines substantially with time apparently respond to canal stimuli but do not use the sensation of G load as an indication that the roll tilt of the gondola or aircraft persists. In contrast, a G-sensitive pilot who misperceives the direction of banking would have neglected the canal input. Also the abrupt transitions in the direction of indicated roll tilt by subject H during a single 2-G period apparently occurred in contradiction with a canal input representing motionlessness. Thus, the identification of these two response patterns supports the notion that humans may have an inherent tendency to prioritize canal or otolith input. Even if not always advantageous for spatial orientation, such a tendency might be more economic, in terms of neural processing, than interpretations based on both sources of information.

### 4.4 Perception of complex motion patterns in centrifuge and aircraft

According to the present findings, as well as those of an earlier study on subjects without flight experience [27], the ability to perceive the bank angle is determined by factors which the aircraft and centrifuge have in common. And this correspondence holds true not only at group level but also if considering individual response characteristics.

#### 4.4.1 Semicircular canal stimuli in roll, yaw and pitch

Nevertheless, the correspondence in perceived roll tilt between the two systems might appear problematic to reconcile with certain earlier findings in the centrifuge. Namely, the magnitude of perceived gondola inclination is dependent not only on the roll-plane angular-*displacement* component per se (the swing out of the gondola) but also on the pattern of the angular-*velocity* components in yaw and pitch, determined by the angular velocity of the centrifuge about its main axle and the subject’s increasing tilt with respect to this axle [21, 25, 26].

An ideal observer, seated upright facing forwards in the gondola, would perceive the initial yaw-left rotation as the entering of a curve, and interpret the increasing sensation of pitch- backward rotation as a consequence of his own change in roll orientation with respect to an Earth-fixed axis of rotation. The roll angular-displacement component would facilitate that interpretation. It has, in fact, been established that the brain’s processing of sensory information on self-motion involves tacit knowledge of physical constraints [13] or estimates of probabilities [11]. Thus, if a subject undergoing acceleration in the centrifuge was capable of regarding the main axis of centrifuge rotation - revealed initially via the yaw stimulus - as an Earth-fixed reference, then the proportions of the yaw and pitch components would convey information about the Earth horizontal plane.

The significance of this source of information is reflected in the fact that the ability to perceive the change in gondola position during acceleration is dependent on the subject’s heading position. In experiments with 8 non-pilots the SVH response was 21±8° in the forward position but only –7±10° degrees when the subjects were facing backwards [21]. In a follow-up study, we compared 8 non-pilots with 9 experienced fighter pilots [26]. In the forward position, the initial SVH tilt was greater in pilots (31±16°) than in non-pilots (17±6°). Also in the backward position the pilots showed a greater initial SVH tilt (–12±17°) than the non-pilots (–5±7°). In both groups the responses were significantly greater in the forward position. Conspicuously, whereas the SVH declined with time (5 min at 2 G) to near-zero in non-pilots, the pilot group showed a negligible decline in the forward position (from 31±16° to 26±23°) but an *increase* when facing backwards (from –12±17 to –24±23°).

#### 4.4.2 Familiarity of the motion pattern as a whole

One explanation for the difference in perceived roll tilt between the forward and backward position is based on concepts adopted from Gestalt psychology [9, 10]. Briefly, when the subject is facing forwards, the pattern formed by the canal stimuli in yaw and pitch would be more familiar; in conjunction with other stimulus components it will contribute to an experience of the motion pattern as a meaningful whole. And if the subject has an adequate imagination of the nature of the motion pattern as a whole, he or she will more likely discern and estimate a single component, e.g. the change in roll position. In contrast, when the subject is facing backwards, the pattern of yaw and pitch angular velocity (i.e. transition from yaw to pitch-forward) would be unfamiliar, which would interfere with the perception of the roll stimulus. Accordingly, the subject’s ability to comprehend the nature of a complex stimulus may be reflected in the perceived magnitude of a single stimulus component [25].

#### 4.4.3 Differences in canal stimuli between aircraft and centrifuge

The significance of the canal stimuli in yaw and pitch for the perception of roll position evokes concerns regarding the possibility of re-creating the entering of a co-ordinated turn by means of a centrifuge-based simulator. Since the radius of a 2-G turn, performed with an aircraft, is much greater than that of a centrifuge, the yaw and pitch components will typically be only about 10 per cent in the aircraft, although the change in roll position can be performed with the same angular velocity as in the centrifuge. Therefore, the contribution of angular velocity in yaw and pitch on the perception of roll tilt makes it seem likely that the perceived bank angle would be smaller during real flight than during a centrifuge run.

Nevertheless, during the entering of a turn with an aircraft, the *proportions* between the yaw and pitch components, i.e. the transition from yaw to pitch-backward, will correspond to the roll tilt in the same way as in the centrifuge. Thus, if the yaw and pitch components are above the stimulus threshold for the canals they will be redundant with the roll plane stimulus and thereby facilitate the perception of a change in roll attitude. This would explain why the SVH tilts were similar in the two systems despite the quantitative difference in the two angular- velocity components. However, since vibrations may increase the perception threshold for canal stimuli, there would be a greater likelihood that the yaw stimulus, indicating the beginning and direction of a turn, will be neglected during certain flight conditions.

#### 4.4.4 The magnitude of the resultant G vector

As regards the increased gravitoinertial force vector, there is no reason to doubt the similarity between a centrifuge run and a real turn with an aircraft. Accordingly, the tendency of pilots to associate an increased sensation of weight with a bank angle was similar in the aircraft as in the centrifuge. However, since the G vector *per se* does not reveal the *direction* of roll tilt, one might consider factors that would make errors in the perceived direction of banking more likely in aircraft. As mentioned, the initial yaw-plane canal stimulus is much weaker in the aircraft, and the pilot’s ability to discern canal stimuli may be impaired by vibrations. Another aspect is that the centrifuge run commences with a jerk and a tangential acceleration, which inevitably alert the pilot, who may become keener to the coming stimulus pattern. Because of such differences it cannot be concluded without further notice that testing in a centrifuge would give a true reflection of the prevalence or significance of this kind of spatial disorientation in aviation.

### 4.5 Practical implications

#### 4.5.1 Quantitative evaluation of simulated flight manoeuvres

No simulation system can reproduce real flight in perfect detail. The evaluation or adjustment of simulation algorithms often involve verbal judgments by experienced pilots. Quantitative recordings of specific components of spatial orientation may be a valuable complement to verbal accounts. The precision of conscious vision, as well as the close connections between vision and the sense of balance, makes a visual indicator particularly useful for the study of spatial orientation. With the contrivance for achieving complete darkness, it can easily be mounted also in an aircraft. The findings of the present experiments strongly indicate that in spite of certain differences between a real co-ordinated turn and a centrifuge run, the perception of roll tilt in pilots does not differ between the two conditions. Thus, the centrifuge may be considered an adequate means for simulating the vestibular stimulus of co-ordinated flight. Issues that remain to be addressed are the significance of neck proprioception and the vestibular effects of head movements. In the present study head movements were avoided (although the head was not completely restrained) whereas during real flight the pilot’s visual scanning of cockpit instruments or of the external environment require frequent head movements. Thus, Coriolis phenomena and G-excess effects [3] may contribute to the disorientation caused by coordinated flight *per se*.

#### 4.5.2 Inter-individual variability

The large inter-individual variability in a group of pilots who had undergone the same strictly scheduled education and were at a similar stage in the career raises questions regarding, on the one hand, underlying learning mechanisms and, on the other hand, practical consequences. One issue is the propensity of pilots to “translate” the sensation of G load into a bank angle. If this ability had been driven by the sensory impressions and demands of ordinary flight, then, the major advantage, in terms of cognitive economy, would not be dependent on quantitative accuracy. Rather, the mere association of increased G load and banking, as a qualitative experience, would serve to maintain the pilot’s awareness of being in a co-ordinated turn.

Nevertheless, the magnitude of perceived bank angle may become a determining factor in rare situations where high cognitive workload or acute psychological stress impairs the pilot’s ability to manoeuvre based on rational scanning of the instruments [1, 24]. Thus, demonstrations in a centrifuge, with recordings of the subjective visual horizontal, may not only provide insight into sensory mechanisms underlying spatial orientation but could also contribute to the pilot’s awareness of personal limitations. As to the question whether the ability to perceive the bank angle during co-ordinated turns can be improved by training in a centrifuge, a basic requirement would be quantitative evaluation also in aircraft.
